# Biomarker selection for detection of occult tumour cells in lymph nodes of colorectal cancer patients using real-time quantitative RT–PCR

**DOI:** 10.1038/sj.bjc.6603206

**Published:** 2006-06-06

**Authors:** L Ohlsson, M-L Hammarström, A Israelsson, L Näslund, Å Öberg, G Lindmark, S Hammarström

**Affiliations:** 1 Department of Clinical Microbiology, Immunology, Umeå University, SE-90185 Umeå, Sweden; 2 Department of Surgery and Perioperative Sciences, Surgery, Umeå University, SE-90185 Umeå, Sweden; 3 Department of Surgery, Helsingborgs Lasarett, Lund University, SE-25187 Helsingborg, Sweden

**Keywords:** carcinoembryonic antigen (CEA), CEA cell adhesion molecule (CEACAM), mucin 2 (MUC2), cytokeratin 20 (CK20), matrix metalloproteinase 7 (MMP7)

## Abstract

Accurate identification of lymph node involvement is critical for successful treatment of patients with colorectal carcinoma (CRC). Real-time quantitative RT–PCR with a specific probe and RNA copy standard for biomarker mRNA has proven very powerful for detection of disseminated tumour cells. Which properties of biomarker mRNAs are important for identification of disseminated CRC cells? Seven biomarker candidates, CEA, CEACAM1-S/L, CEACAM6, CEACAM7-1/2, MUC2, MMP7 and CK20, were compared in a test-set of lymph nodes from 51 CRC patients (Dukes' A–D) and 10 controls. Normal colon epithelial cells, primary tumours, and different immune cells were also analysed. The biomarkers were ranked according to: (1) detection of haematoxylin/eosin positive nodes, (2) detection of Dukes' A and B patients, who developed metastases during a 54 months follow-up period and (3) identification of patients with Dukes' C and D tumours using the highest value of control nodes as cutoff. The following properties appear to be of importance; (a) no expression in immune cells, (b) relatively high and constant expression in tumour tissue irrespective of Dukes' stage and (c) no or weak downregulation in tumours compared to normal tissue. CEA fulfilled these criteria best, followed by CK20 and MUC2.

It is now well established that the earlier the diagnosis of cancer, the better the clinical outcome, through earlier administration of effective and possibly curative treatments (Etzioni *et al*, 2003). For colorectal cancer (CRC) the only curative treatment is surgery. However, even after curative surgery the tumour recurs in many instances. In CRC the prognostic predictor is tumour stage based on histopathologic examination of the resected specimen combined with perioperative findings (Dukes and Bussey, 1958; Lindmark *et al*, 1994; Fleming *et al*, 1997). It has been shown that adjuvant chemotherapy reduces the relative mortality rate by one-third in patients operated for colon cancer in Dukes' Stage C (any TN1-2M0, Stage III) (Moertel *et al*, 1995; Fleming *et al*, 1997). Adjuvant chemotherapy is not routinely given to patients of Dukes' Stage A (T1-2N0M0, Stage I) or Stage B (T3-4N0M0, Stage II) although a substantial number of patients with Dukes' Stage B tumour will die from tumour recurrences. It is considered to be of vital importance to improve the selection criteria in identifying patients who may benefit from adjuvant chemotherapy and intense follow-up protocols.

A key to these efforts is finding ways to specifically identify disseminated tumour cells in regional lymph nodes. Different techniques, for example analysis of multiple haematoxylin/eosin (H&E)-stained sections, immunohistochemistry, gel-based qualitative reverse transcriptase–polymerase chain reaction (RT–PCR) and more recently real-time quantitative RT–PCR (qRT–PCR), and biomarkers including so-called surrogates of cancer have been utilised (Tsavellas *et al*, 2001; Lotze *et al*, 2005). It can now be concluded that real-time qRT–PCR assay for biomarker mRNA, particularly when used with a specific probe and RNA copy standard, is a superior method for micrometastases detection because it is objective, highly sensitive and quantitative, and has a very wide measuring range (Godfrey *et al*, 2001; Ho *et al*, 2004; Öberg *et al*, 2004). Moreover, this technology lends itself to automation and rapid test performance suitable for a clinical setting (Raja *et al*, 2005).

In a recent study, we used real-time qRT–PCR for detection of disseminated tumour cells in lymph nodes of CRC patients using mRNA for two commonly used tumour markers, carcinoembryonic antigen (CEA) and cytokeratin 20 (CK20) (Öberg *et al*, 2004). The results were promising, particularly for CEA. However, there might be biomarkers even more suitable for this purpose and neither CEA nor CK20 discriminates between dislocated normal intestinal epithelial cells (iECs) and tumour cells with propensity to metastasize. Three kin molecules to CEA are expressed in the colonic epithelium, that is CEA cell adhesion molecule-1 (CEACAM1), CEACAM6 and CEACAM7 (Horst and Wagener, 2004; Frängsmyr *et al*, 1995, 1999). CEACAM6 has a wider cellular distribution than CEA. However, it has been claimed that tumour levels are higher for CEACAM6 than CEA and that CEACAM6 therefore would be the better tumour marker (Ilantzis *et al*, 2002; Jantscheff *et al*, 2003). CEACAM1 comes with a long (CEACAM1-L) or short (CEACAM1-S) cytoplasmic tail. The long cytoplasmic tail of CEACAM1-L was shown to mediate tumour suppressor function in model systems while the short cytoplasmic tail of CEACAM1-S lacks this property (Hammarström, 1999; Horst and Wagener, 2004). Thus, CEACAM1-S might be a useful biomarker and an increased CEACAM1-S/CEACAM1-L ratio an indicator of tumour transformation. As far as one knows, CEACAM7 shows the same restricted expression pattern as CEA. CEACAM7 mRNA comes in two splice forms (CEACAM7-1 and CEACAM7-2) coding for molecules that differ in the number of extracellular immunoglobulin-like domains. Little is known about their relative expression levels and possible changes in CRC. Mucin 2 (MUC2) is the major mucin in colon (Weiss *et al*, 1996). It is a candidate tumour marker since its expression is restricted to epithelial cells. Finally, matrix metalloproteinase 7 (MMP7)/matrilysin is an interesting candidate biomarker since it is expressed in the invasive front of the primary tumour and MMP7 expression is correlated with capacity to form metastases in CRC transfer experiments (Adachi *et al*, 2001; Yamamoto *et al*, 2003).

The aims of this study were: (1) to determine which properties of a tumour mRNA marker decide if it is suitable for detection of tumour cells in tissues with an excess of immune cells, (2) to find tumour markers that would be complementary to CEA mRNA by improving sensitivity for the identification of disseminated tumour cells in lymph nodes of CRC patients and (3) to find a biomarker for tumour cells with metastasizing capacity.

## MATERIALS AND METHODS

### Patients

Surgery for CRC was carried out in 51 patients (32 men, 19 women; median age 69 years, range 52–90). Thirty-five tumours were located in colon and 16 in rectum. Seven patients with rectal cancer received preoperative irradiation with 25 Gy. Radical excision of the tumours with wide lymph node dissection was carried out in 42 patients. Nine patients had distant metastases. Five patients received adjuvant chemotherapy. According to Dukes' classification there were six tumours in Stage A (T1-2N0M0, Stage I), 26 in Stage B (T3-4N0M0, Stage II), 10 in Stage C (anyTN1-2M0, Stage III) and nine in Stage D (anyTanyNM1, Stage IV). At follow-up after median 54 months (range: 35–68) 19 patients had died from CRC and eight patients had died from noncancer disease. None of the living patients had tumour recurrence.

Controls included seven men and three women (median age 30 years, range: 18–61) undergoing colorectal surgery for ulcerative colitis (UC; *n*=6), Crohn's disease (CD; *n*=3) and rectal prolapse (*n*=1). Informed consent was obtained from the patients. The local Research Ethics Committee of the Medical Faculty, Umeå University, Sweden, approved our study.

### Lymph nodes

One to four lymph nodes were dissected from surgically removed specimens and bisected with separate knives under sterile conditions to prevent RNA cross-contamination. One half of each node was fixed in 10% buffered formalin for routine H&E staining and the other half was snap frozen in liquid nitrogen and stored at −70°C until RNA extraction. In all, 95 lymph nodes were collected from the CRC patients (11 nodes from Dukes' Stage A patients, 55 nodes from Dukes' Stage B patients, 16 nodes from Dukes' Stage C patients and 16 nodes from Dukes' Stage D patients). In the control group 18 nodes were from UC patients, 12 nodes from CD patients and four nodes from the patient with rectal prolapse.

### CRC tissue

An approximately 0.5 × 0.5 × 0.5 cm piece was collected from the outer rim of 20 tumour specimens immediately after resection (two Dukes' Stage A, 13 Dukes' Stage B, two Dukes' Stage C and three Dukes' Stage D), snap-frozen and kept at −70°C until RNA extraction.

### Epithelial cells from colon tissue

Colonic epithelial cells were isolated from apparently normal tissue constituting the resection margin after surgical removal of tumour in CRC patients and from colon of UC patients subjected to surgical treatment as described earlier (Fahlgren *et al*, 2003b). The isolation procedure yields one fraction enriched in crypt epithelial cells (crypt-iECs) and one fraction enriched in luminal epithelial cells (luminal-iECs).

### Cell lines and peripheral blood mononuclear cells

Human cell lines used were: LS174T, T84, HT29, and HCT8 (colon carcinomas), Jurkat and Molt-4 (T-cell lymphomas), CNB6 and KR4 (EBV-transformed B cell lines; a mixture of equal amounts of RNA from the two lines was used in the analyses), U266 (plasmacytoma), U937 (monocyte-like cell line), K562 (erythroblastoid cell line), HL60 (promyelocytic cell line). Peripheral blood mononuclear cells (PBMC) were isolated from peripheral blood of healthy adults by Ficoll-Isopaque gradient centrifugation. PBMC were *in vitro* activated by incubation with anti-CD3 mAb OKT3 (50 ng ml^−1^) in HEPES-buffered RPMI1640 supplemented with 0.4% human serum albumin. PBMC from seven individuals were incubated with the stimulus in parallel cultures for 4, 7, 20, 48 and 72 h, washed, pooled and RNA extracted.

### RNA extraction

Total RNA was extracted using the Acid Guanidine Phenol Chloroform (AGPC) method by adding 0.5 ml of a solution containing 4 M guanidinium thiocyanate, 25 mM sodium citrate (pH 7), 0.5% sarcosyl and 0.1 M 2-mercaptoethanol per 25 mg tissue and up to 2.5 × 10^6^ cells in the first homogenization step. Extracted RNA was dissolved in RNAse-free water containing RNAse inhibitor.

### Real-time qRT–PCR

Real-time qRT–PCR assays for CEA, CEACAM6, CEACAM1-S, CEACAM1-L, CEACAM7-1, CEACAM7-2, MUC2, MMP7 and CK20 mRNAs were constructed in the laboratory using the TaqMan EZ technology (Applied Biosystems, Foster City, CA, USA). Specific primer pairs were placed in different exons and a dye-labelled probe was placed over the boundary between the two exons in the amplicon. Assays for all markers except MMP7 have been described (Fahlgren *et al*, 2003a; Forsberg *et al*, 2004; Öberg *et al*, 2004). The sequences for the MMP7 primers and probe were: forward primer 5′-GGGAGGCATGAGTGAGCTAC-3′, reverse primer 5′-TCTCCTTGAGTTTGGCTTCTAAA-3′ and probe 5′-TCTTGAGATAGTCCTGAGCCTGTTCCCA-3′. The reporter dye at the 5′-end of each probe was FAM. The quencher dye at the 3′-end was TAMRA for CEA, CEACAM1-L, CEACAM6, CEACAM7-2, CK20 and MMP7, and MGB for CEACAM1-S, CEACAM7-1 and MUC2. Emission from released reporter dye was monitored by the ABI Prism 7700 Sequence Detection System (Perkin-Elmer, Wellesley, MA, USA). The RT–PCR profile for all assays except CEACAM7-1 was: 49°C for 2 min, 59°C for 30 min, 94°C for 5 min followed by 45 cycles of 93°C for 20 s and 61°C for 1 min. The RT–PCR profile for CEACAM7-1 was: 49°C for 2 min, 59°C for 30 min, 94°C for 5 min followed by 45 cycles of 93°C for 20 s and 59°C for 1 min. Specific RNA copy standards were prepared as described previously (Fahlgren *et al*, 2003b). Determinations were carried out in triplicates and expressed as copies of mRNA per *μ*l as determined from parallel RT–PCR of serial dilutions of the RNA copy standard. The concentration of 18S rRNA was determined in each sample by real-time qRT–PCR according to the manufacturer's protocol (Applied Biosystems). As no copy standard is available for the 18S rRNA assay the 18S rRNA content was expressed as arbitrary units defined as the amount of 18S rRNA in 1 pg total RNA extracted from PBMC. Results are expressed as mRNA copies per unit of 18S rRNA.

### Statistics

The mRNA expression levels in iECs of normal and inflamed colon were compared using two-tailed Mann–Whitney's rank sum test. Comparisons between mRNA expression levels in tumour *vs* normal iECs and between levels of different mRNA species in tumour tissue were performed using Kruskal–Wallis' nonparametric one-way ANOVA with Dunn's multiple comparison *post hoc* test. Analyses of correlations between levels of different mRNA species were performed using two-tailed Spearman rank correlation test. A *P*-value <0.05 was considered statistically significant.

## RESULTS

### Biomarker mRNA levels in colonic epithelial cells and CRC tumours

Levels of CEA, CEACAM1-S/L, CEACAM6, CEACAM7-1/2, MUC2, MMP7 and CK20 mRNAs were assessed in RNA extracted from primary tumours representing all four Dukes' stages and from luminal-iECs and crypt-iECs of apparently normal and inflammatory colon using specific real-time qRT–PCR assays with RNA copy standards. The 18S rRNA concentration was determined in each sample and levels are expressed as mRNA copies/18S rRNA unit allowing direct comparison between the different biomarkers. Statistically significant difference in mRNA levels between iECs from normal and inflammatory colon was only seen for MMP7 (see legend to Table 1), therefore the two groups were combined in the comparisons against tumour tissue. Table 1 summarises the results. The mRNA levels of the different biomarkers varied considerably within the tumours, from median 107 mRNA copies/18S rRNA unit for CEA to 0.05 for CEACAM7-1. CEA and CEACAM6 mRNAs were expressed at approximately equal levels in tumours and iECs while the mRNA levels for CEACAM7-1/2, CEACAM1-L, MUC2 and CK20 were significantly decreased in the tumour. Only MMP7 mRNA levels were increased in the tumour compared to iECs (Table 1). Table 2 shows the mRNA levels of the biomarkers in four CRC cell lines. Among the biomarkers CEA exhibited the highest expression level and smallest variation between CRC cell lines.

### Biomarker mRNA levels in immune cells

Table 2 summarises the results of analysis of polyclonally activated and resting PBMC and seven immune cell lines. Four biomarker mRNAs were not detected or were detected in trace amounts only in one type of immune cells. These were MUC2, CEA, CEACAM7-2 and CK20. Other biomarkers were expressed at high levels in one or several types of immune cells (CEACAM1-L, CEACAM6, MMP7) or at low levels (CEACAM1-S, CEACAM7-1).

### Specificity indexes

To rank the different biomarkers with respect to specificity for CRC we calculated two types of specificity indexes: (I) median value in CRC tumours/highest value in any type of immune cell and (II) median value in CRC tumours/highest value of control lymph nodes (Table 3). As can be seen, the two indexes gave similar values with the exceptions of those for CEACAM6 and CEACAM7-2. CEA was the most specific biomarker followed by CK20. CEACAM6 showed better specificity with index II than index I, while the reverse was true for CEACAM7-2. Indexes for three biomarkers (CEACAM1-L, CEACAM7-1, MMP7) showed no specificity for CRC.

### Biomarker mRNA levels in lymph nodes of CRC patients and controls

**Figure 1:**
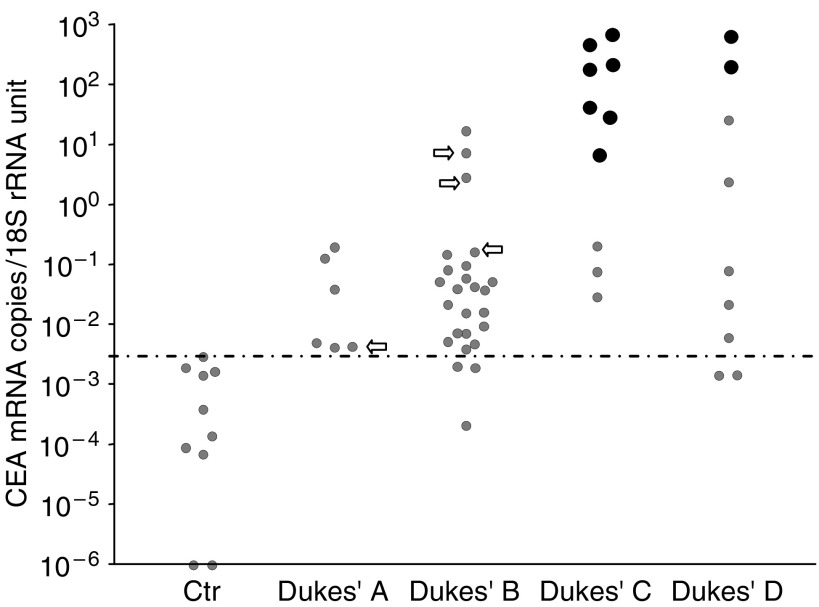
CEA mRNA levels in lymph nodes as determined by real-time qRT–PCR for CEA mRNA and 18S rRNA content in the samples. Each patient is represented by the lymph node with the highest CEA mRNA level (dots). Arrows indicate the four patients with Dukes' Stage A and B tumours, who had died from CRC during the 54 months (range: 35–68 months) follow-up time. Large black dots indicate lymph nodes that had tumour cells identified by H&E staining and small grey dots indicate H&E negative nodes. Results updated from Öberg *et al*, 2004.

**Figure 2:**
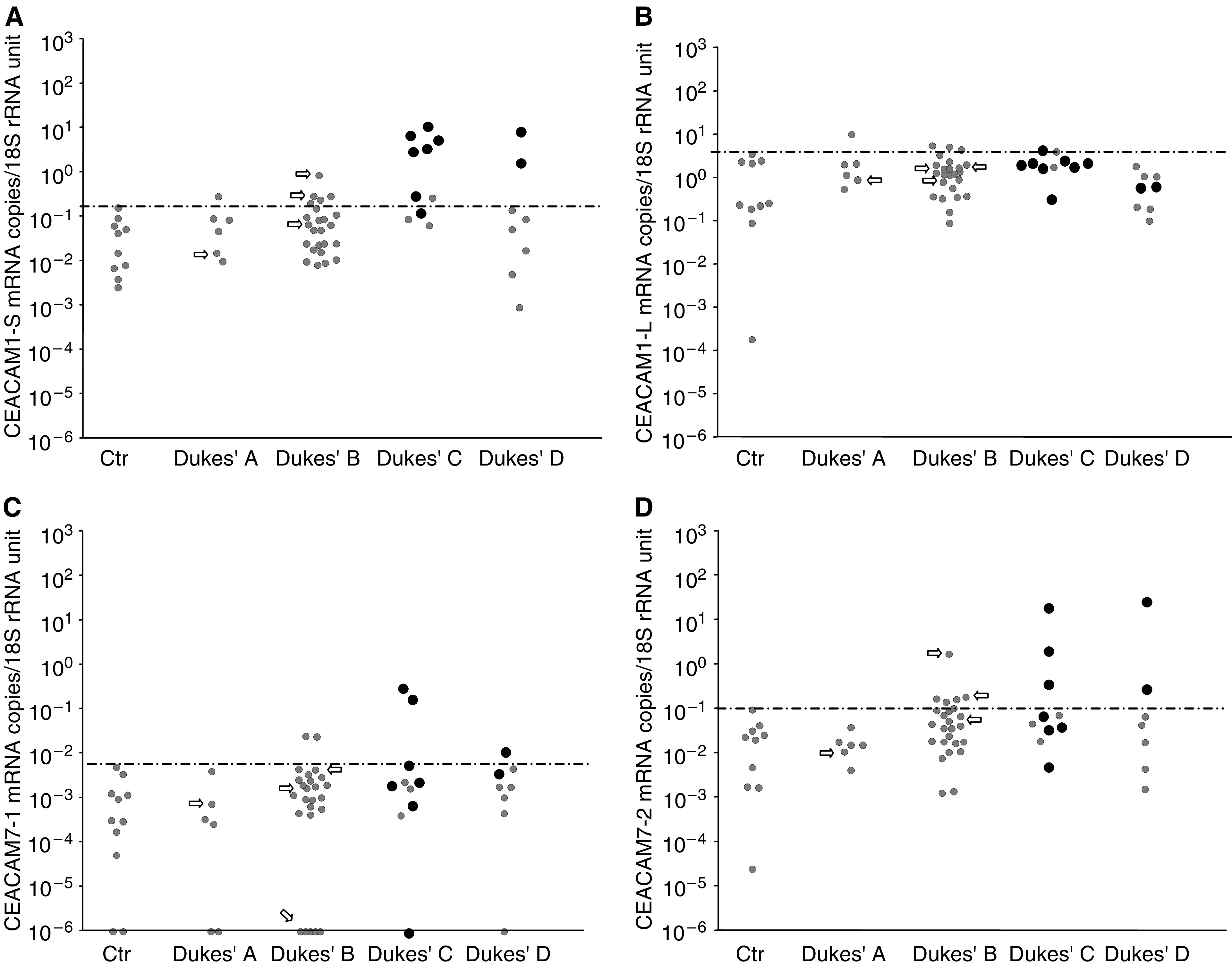
CEACAM1-S (**A**), CEACAM1-L (**B**), CEACAM7-1 (**C**), and CEACAM7-2 (**D**) mRNA levels in lymph nodes of CRC and control patients. Each patient is represented by the lymph node with the highest level of the respective mRNA species. For explanation of dots and arrows see legend to Figure 1.

**Figure 3:**
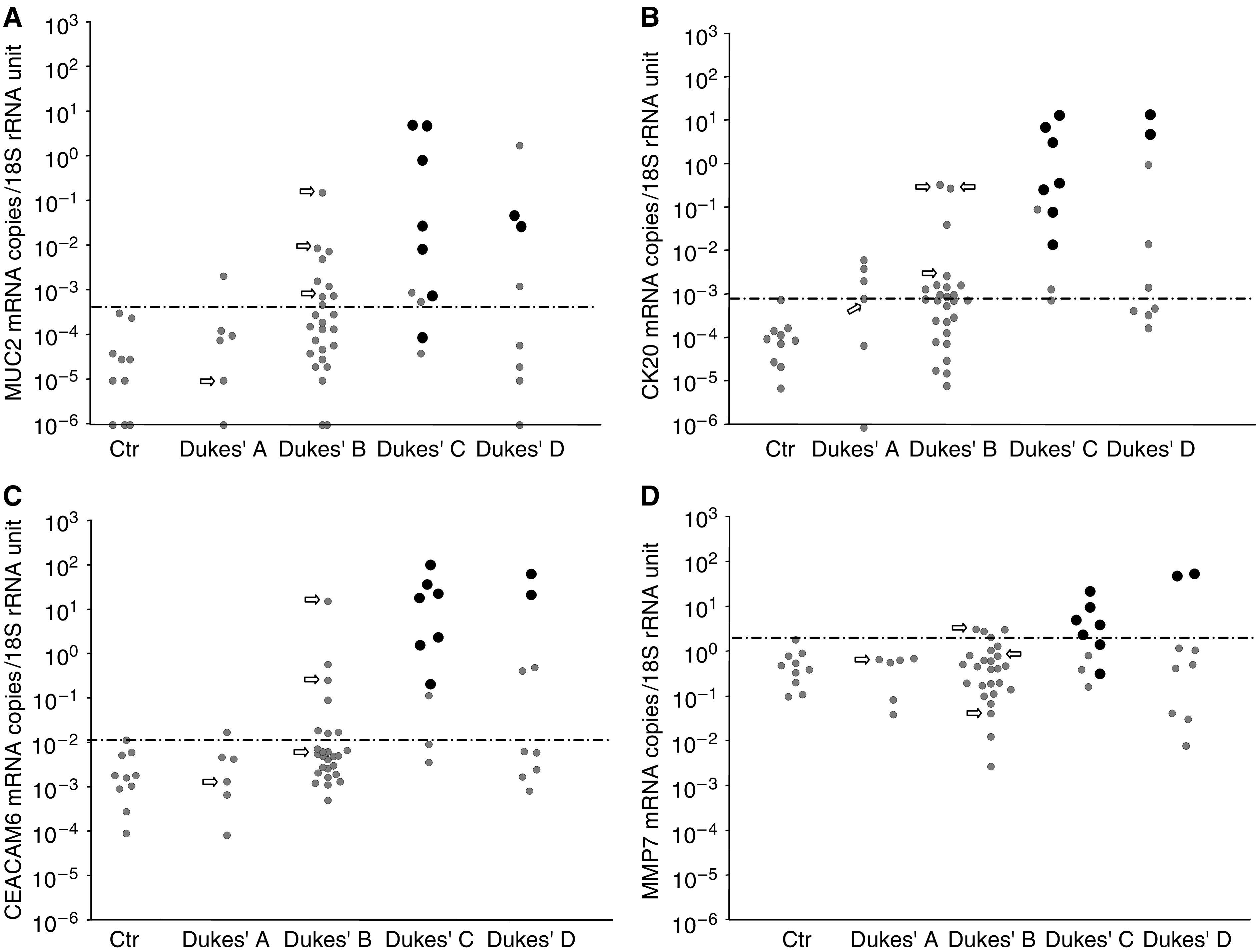
MUC2 (**A**), CK20 (**B**), CEACAM6 (**C**), and MMP7 (**D**) mRNA levels in lymph nodes of CRC and control patients. Each patient is represented by the lymph node with the highest level of the respective mRNA species. For explanation of dots and arrows see legend to Figure 1.

A total of 129 lymph nodes of 51 CRC patients and 10 controls were analysed for mRNA levels of the biomarkers. Figures 1, 2 and 3 summarise the results. For each individual only the lymph node with the highest level of the indicated mRNA species is shown. For each biomarker, a cutoff level is set at the highest control node.

CEA mRNA showed excellent discrimination between nodes from CRC patients and controls (Figure 1). Practically all CRC nodes were above the cutoff level independent of Dukes' Stage and all H&E positive lymph nodes had very high CEA mRNA levels. Moreover, the three Dukes' B patients that have died from their CRC within the 54 months follow-up period displayed highly elevated CEA mRNA levels (arrows in figure). The single Dukes' A patient that has died from her CRC showed a borderline value (arrow).

CEACAM1-S mRNA (Figure 2A) showed some discrimination between CRC and controls. Nodes of 1/6 Dukes' A, 5/24 Dukes' B, 7/10 Dukes' C and 2/8 Dukes' D patients were above cutoff and all but one H&E positive nodes were identified as positive by CEACAM1-S mRNA level. Moreover, two of the three Dukes' B patients, but not the Dukes' A patient, that have died from their CRC within the follow-up period were identified. Note also that the total range of values is much smaller for CEACAM1-S than for CEA, that is four and seven orders of magnitude, respectively, and that the CEACAM1-S levels in controls are almost 100 times higher.

No discrimination between CRC and control nodes was seen with CEACAM1-L mRNA (Figure 2B). The CEACAM1-L mRNA levels of control nodes were very high, which is consistent with the high levels of CEACAM1-L mRNA in immune cells (Table 2).

CEACAM7-1 mRNA was not a useful marker (Figure 2C) while CEACAM7-2 mRNA, which was expressed at about 100 times higher concentration, displayed some discriminating power between CRC patients and controls (Figure 2D). However, several H&E positive nodes were missed and one of the three Dukes' B patients and the Dukes' A patient that have died from CRC within the follow-up period were missed.

MUC2 mRNA showed good discrimination between CRC nodes and control nodes comparable to that of CEA mRNA. All but one of the H&E positive nodes and all three Dukes' B patients who had died from CRC had values above the cutoff (Figure 3A). Interestingly, MUC2 mRNA values are very low in controls and the range of values in CRC is quite large.

Similarly, CK20 mRNA showed good discrimination between nodes from CRC patients and controls (Figure 3B). All H&E positive nodes as well as the three Dukes' B patients who have died from CRC during the follow-up period were identified as positive. CK20 mRNA levels in CRC nodes varied over a wide range and control nodes were low.

CEACAM6 mRNA displayed fairly good ability to discriminate between CRC patients and controls (Figure 3C). However, only two of the three Dukes' B patients that have died from their CRC within the follow-up period had elevated CEACAM6 levels. All H&E positive nodes displayed CEACAM6 mRNA levels above cutoff.

MMP7 mRNA displayed poor discriminating power between CRC patients and controls (Figure 3D).

### Correlation between different biomarker mRNA levels in lymph nodes of CRC patients and controls

The four biomarker mRNAs which showed the best discriminating power between CRC and control nodes and which were not appreciably expressed in immune cells namely CEA, CEACAM7-2, CK20 and MUC2 were analysed by pair-wise comparisons of biomarker levels in all 129 lymph nodes. Each Dukes' Stage and controls were also analysed separately. Table 4 shows correlation coefficients (*r*-values) and significance levels (*P*-values) for all six comparisons. Biomarker mRNA values were significantly correlated if all nodes were compared with *r*-values ranging from 0.47 to 0.76. When the different stages of CRC were analysed separately it was noted that lymph nodes from Dukes' C and Dukes' D patients showed *r*-values⩾0.5 and *P*-values⩽0.05 for all marker combinations except one indicating that it was the same cells that expressed the four markers. The comparisons furthermore demonstrate that CEACAM7-2 is also expressed in a separate cell population normally present in lymph nodes. The results also indicate that control nodes harbour low numbers of two cell populations of epithelial origin, one expressing CEA, CK20 and CEACAM7-2 and another expressing MUC2, CK20 and CEACAM7-2.

## DISCUSSION

In this study, we have explored the possibility of using real-time qRT–PCR as an alternative, or adjunct, to the classical H&E staining method for detection of disseminated tumour cells in regional lymph nodes. There are several reasons to search for alternatives. Firstly an inherent problem with histochemical as well as immunohistochemical methods is that of sampling. Usually, only one or a few 6 *μ*m thick sections are analysed covering <1% of the lymph node volume. To analyse the number of sections that would be needed to properly cover the node becomes impossible in practice. In the qRT–PCR study reported here, we extracted RNA from one half of the lymph node. A second problem specific for H&E is to decide on the basis of morphology alone whether a small number of cells indeed are tumour cells. Thirdly, and in contrast to H&E and immunohistochemistry, the qRT–PCR assay is very sensitive and objective and has a very large measuring range. With the appropriate choice of primers and probes, it can be made highly specific for the chosen marker mRNA. Moreover, since analyses are performed at the mRNA level instead of at the protein level positive results are likely to reflect the presence of living tumour cells. It has been shown that dendritic cells/macrophages can transport protein components from apoptotic epithelial cells to lymph nodes (Bonnotte *et al*, 2000; Huang *et al*, 2000). A yet unresolved question in using qRT–PCR for the purpose of detecting disseminated tumour cells in regional lymph nodes is, however, which biomarker mRNA to use.

A major goal of this study was to determine which properties of a tumour marker mRNA are of importance for the detection of disseminated colon tumour cells in the presence of an excess of immune cells for example in regional lymph nodes and blood. We, therefore, investigated a number of biomarkers, that are expressed in colon adenocarcinoma cells and compared their expression levels in a test-set of lymph nodes from CRC patients of different Dukes' stages and controls as well as in primary tumours, in epithelial cells of normal and inflamed colon and in different types of immune cells.

We found that CEACAM1-S/L, CEACAM7-1/2, MUC2 and CK20 mRNAs were expressed at lower levels in the primary tumour compared to normal colon. CEACAM1 and CEACAM7 mRNAs have previously been shown to be downregulated in CRC using semiquantitative methods (Neumaier *et al*, 1993; Nollau *et al*, 1997). In contrast, neither CEA nor CEACAM6 mRNA levels were decreased in tumour tissue. MMP7 was the only marker that exhibited increased levels in tumours. However, the levels were still low compared to CEA. CEA and CEACAM1-S levels displayed the smallest variation between individual primary tumour tissue samples and there was no tendency for changes in relation to Dukes' stages for these two markers. Of all biomarkers CEA mRNA was expressed at the highest levels in tumours followed by CEACAM6. The high levels of these two markers could possibly be explained by the fact that CEA and CEACAM6 are expressed both in columnar epithelial cells and goblet cells in normal colon while CEACAM1 and CEACAM7 are expressed in columnar epithelial cells only and MUC2 in goblet cells only (Weiss *et al*, 1996; Frängsmyr *et al*, 1999).

It can be argued that no or only marginal expression of the biomarker in immune cells should be of utmost importance for successful detection of tumour cells in lymph nodes. Indeed, three of the four biomarkers that were expressed in only trace amounts in immune cells, that is CEA, CK20 and MUC2, showed good discriminatory capacity between nodes from CRC patients and controls. Owing to its low expression in immune cells one would have predicted excellent discriminatory capacity also by CEACAM7-2 but this was not the case. Possibly CEACAM7-2 is expressed in some additional cell type in lymph nodes, for example endothelial cells or stromal cells.

CEA mRNA showed excellent separation between CRC patients and controls. MUC2 and CK20 mRNAs had almost the same discriminating power as CEA mRNA. The Dukes' B patients who developed metastatic disease during the follow-up period were all detected by the three markers but only for CEA were all Dukes' C nodes above the cutoff value. Moreover, one H&E positive lymph node was missed by the MUC2 mRNA assay.

It was somewhat unexpected that MUC2 gave essentially the same result as CEA although in normal colon MUC2 is confined to goblet cells and not expressed in columnar epithelial cells. Does all CRC tumour disseminated to the lymph node contain goblet cell-like elements? We do not know whether this is the case. Possibly MUC2 expression can be induced by external agents in CRC tumour cells. It was recently shown that bile acids induce MUC2 overexpression in human colon carcinoma cells (Song *et al*, 2005). Furthermore, we found that the inflammatory state of the small intestinal mucosa in patients with active celiac disease is associated with ectopic production of MUC2 by enterocytes (Forsberg *et al*, 2004). Thus, the cytokine milieu in the nodes might upregulate MUC2 expression in the iECs.

Although CEA and CEACAM6 behaved very similar CEA had a higher discriminatory power. This was due to CEA that, contrary to expectation, had higher expression level in tumour cells than CEACAM6 and that CEACAM6 was expressed in myeloid immune cells with the risk that tumour cells expressing fairly low levels of this marker would drown in the immune cell background of the node.

The two markers for tumour cell ‘aggressiveness’ studied here, that is MMP7 and CEACAM1-S, were not suitable for analysis of lymph nodes. MMP7 because its expression was induced in activated T lymphocytes, a cell type which is likely to be prominent in lymph nodes. CEACAM1-S was not suitable because (1) it was expressed in several types of immune cells although at low levels and (2) the very high expression level of CEACAM1-L in immune cells precludes meaningful calculation of CEACAM1-S: CEACAM1-L ratios in lymph nodes.

In conclusion, the finding that three independent biomarker mRNAs, CEA, MUC2 and CK20, gave almost the same results with highly selective expression in cells of epithelial origin strongly support the notion that disseminated tumour cells can be successfully detected in regional lymph nodes by this technique. CEA mRNA appears to be the best choice as single marker due to its remarkably high expression level in colorectal tumour cells.

## Figures and Tables

**Table 1:** Expression levels of mRNAs for CEA, CEACAM1, CEACAM6, CEACAM7, MUC2, MMP7, and CK20 in primary colorectal tumours compared to control colonic epithelial cells

	**Tumour**		**Crypt iEC^a^**			**Luminal iEC**		
**mRNA species**	**Median^b^**	**IQR^c^**	**Median**	**IQR**	***P*-value^d^**	**Median**	**IQR**	***P*-value**
CEA	107	39–1207	261	225–537	NS	393	212–769	NS
CEACAM6	20	6.5–55	28	14–35	NS	38	13–116	NS
CEACAM1-S	5.1	2.5–14	19	12–34	<0.01	28	12–72	<0.01
CEACAM1-L	1.4	0.43–6.4	16	8.0–40	<0.001	22	10–40	<0.001
CEACAM7-1	0.05	0.005–0.37	0.90	0.67–2.9	<0.001	1.9	1.2–6.0	<0.001
CEACAM7-2	9.0	0.94–32	302	162–468	<0.001	483	361–582	<0.001
MUC2	0.66	0.04–3.4	32	25–56	<0.001	33	17–77	<0.001
MMP7	1.7	0.85–4.3	0.09	0.01–0.25	<0.01	0.09	0.003–16	<0.05
CK20	14	5.5–40	295	178–675	<0.001	162	104–377	<0.001

a iEC=intestinal epithelial cells.

b Median mRNA copies/18S rRNA unit of 20 CRC tumour samples (2 Dukes' Stage A, 13 Dukes' Stage B, 2 Dukes' Stage C and 3 Dukes' Stage D), 14–20 crypt iEC samples and 10–20 luminal iEC samples. Equal numbers of iEC samples were derived from the apparently healthy resection margin of colon from patients operated for CRC and from colon of UC patients. The mRNA expression levels for any of the markers in iEC from normal colon of CRC patients and UC colon, respectively, did not differ significantly from each other with the exception of MMP7 mRNA. MMP7 mRNA levels were significantly higher (*P*<0.01) in the iECs from UC patients compared to iEC from the resection margin of CRC patients. MMP7 values for iEC in the Table include data from 10 CRC and 10 UC patients.

c IQR=interquartile range from the 25 to the 75 percentile.

d *P*-value obtained by comparing mRNA expression levels in tumours with those in crypt iECs, and luminal iECs using Kruskal–Wallis nonparametric one-way ANOVA with Dunn's multiple comparison *post hoc* test. NS=not significant, that is *P*-value >0.05.

**Table 2:** Expression levels of mRNA for CEA, CEACAM1, CEACAM6, CEACAM7, MUC2, MMP7, and CK20 in CRC cell lines and different types of immune cells

		**Biomarker mRNA**								
**Cells**	**Origin**	**CEA**	**CEACAM6**	**CEACAM1-S**	**CEACAM1-L**	**CEACAM7-1**	**CEACAM7-2**	**MUC2**	**MMP7**	**CK20**
HT29	Colon iEC	32^a^	43	22	17	0.003	0.3	0.01	53	85
LS174T	Colon iEC	328	81	1.2	1.4	0.5	38	4.3	3.4	0.02
T84	Colon iEC	33	1.0	0.9	0^b^	0.003	0.07	0.5	2.3	33
HCT8	Colon iEC	32	0.5	0.003	0	0	0.1	0.02	0.2	0.05

PBMC		0	0.05	0	0.3	0.01	0	0	0.001	0
Act. PBMC		0	0	0.001	0.8	0	0	0	2.0	0
Jurkat	T cell	0	0	0.004	0.1	0	0	0	0.004	0
Molt-4	T cell	0	0	0.002	0.05	0	0	0	0	0
CNB6+KR4	B cell	0	0	0.008	1.1	0.05	0	0	0.6	0
U266	Plasma cell	0	0	0.001	0.02	0	0	0	0	0
U937	Monocyte	0.005	0.3	0.002	0.03	0	0	0	0.9	0.003
HL60	Granulocyte	0	1.8	0	ND	0	0.001	ND	0	0
K562	Pre-erythrocyte	0	0	0.006	0.08	0	0	0	0	ND

a Expression level of the indicated mRNA species expressed as mRNA copies/18S rRNA unit.

b Values below 0.001 mRNA copies/18S rRNA unit are recorded as 0.

ND=not determined.

**Table 3:** Specificity indexes for tumour marker mRNAs

**mRNA species**	**Median CRC tumour value/highest value of any immune cell type**	**Median CRC tumour value/highest value of control lymph nodes**
CEA	21 400	53 500
CEACAM6	11	2000
CEACAM1-S	638	39
CEACAM1-L	1.3	0.4
CEACAM7-1	1.0	6.3
CEACAM7-2	9000	90
MUC2	>660	942
MMP7	0.9	1.1
CK20	4667	15 555

**Table 4:** Correlation between expression levels of biomarker CEA, CEACAM7-2, CK20 and MUC2 mRNAs in lymph nodes of CRC patients and controls

	**All lymph nodes**		**Controls**		**Dukes' A**		**Dukes' B**		**Dukes' C**		**Dukes' D**	
**Compared mRNA species**	***r* a**	***P*-value^a^**	***r***	***P*-value**	***r***	***P*-value**	***r***	***P*-value**	***r***	***P*-value**	***r***	***P*-value**
CEA *vs* CK20	0.76	<0.0001	0.47	0.005	0.80	0.005	0.58	<0.0001	0.70	0.004	0.76	0.0006
MUC2 *vs* CK20	0.62	<0.0001	0.50	0.004	0.62	0.04	0.45	0.001	0.69	0.007	0.60	0.05
CEA *vs* MUC2	0.71	<0.0001	0.33	NS	0.60	NS	0.55	<0.0001	0.78	0.0006	0.80	0.003
CEA *vs* CEACAM7-2	0.62	<0.0001	0.64	<0.0001	0.42	NS	0.40	0.004	0.54	0.03	0.89	0.0003
CEACAM7-2 *vs* CK20	0.47	<0.0001	0.53	0.001	0.37	NS	0.16	NS	0.52	0.05	0.85	0.002
CEACAM7-2 *vs* MUC2	0.55	<0.0001	0.43	0.02	0.44	NS	0.43	0.003	0.41	NS	0.80	0.005

a *r*- and *P*-values obtained by pair-wise comparing the expression levels of the indicated mRNA species in lymph nodes of CRC patients and controls using two-tailed Spearman rank correlation test. Numbers of lymph nodes in the analysis were 120–129 for all lymph nodes, 33–34 for controls, 10–11 for Dukes' A patients, 48–52 for Dukes' B patients, 15–16 for Dukes' C patients and 12–16 for Dukes' D patients. NS=not significant, *P*-value>0.05.
